# Epidemiology of Malaria in East Nusa Tenggara Province in Indonesia: Protocol for a Cross-sectional Study

**DOI:** 10.2196/23545

**Published:** 2021-04-09

**Authors:** Robertus Dole Guntur, Jonathan Kingsley, Fakir M Amirul Islam

**Affiliations:** 1 Department of Health Science and Biostatistics School of Health Sciences Swinburne University of Technology Melbourne Australia; 2 Department of Mathematics Faculty of Science and Engineering Nusa Cendana University Kupang NTT Indonesia; 3 Department of Health and Medical Sciences School of Health Sciences Swinburne University of Technology Melbourne Australia; 4 Centre of Urban Transitions Swinburne University of Technology Hawthorn Melbourne Australia

**Keywords:** malaria, rural population, awareness, risk factors, health policy, World Health Organization

## Abstract

**Background:**

Malaria is a global pandemic that results in approximately 228 million cases globally; 3.5% of these cases are in Southeast Asian countries, including Indonesia. Following the World Health Organization (WHO) initiative, Indonesia is in the process of achieving malaria-free zone status by 2030. However, the eastern part of Indonesia, including the East Nusa Tenggara Province (ENTP), still has a disproportionately high rate of malaria.

**Objective:**

The aims of this cross-sectional study are to determine the awareness and knowledge, attitude, and practice toward various aspects of malaria among rural adults and their associated factors, including sociodemographic factors and ethnicities; assess the gap between coverage of, access to, and use of long-lasting insecticide-treated nets (LLINs) among the households; estimate the prevalence of and factors associated with malaria in rural adults; and develop a risk prediction model for malaria.

**Methods:**

A multistage cluster sampling procedure with a systematic random sampling procedure at cluster level 4 was applied to recruit 1503 adults aged 18 years or older from the ENTP. Each participant participated in a face-to-face interview to assess their awareness and knowledge, attitude, and practice toward aspects of malaria, practices of sleeping under LLINs, and history of malaria. Information on sociodemographic, environmental, and lifestyle factors was also documented. The proportion of knowledge, attitude, and practice toward aspects of malaria and their variations across different sociodemographic and ethnic groups will be analyzed using descriptive statistics and chi-square tests. Coverage and access to LLINs will be evaluated based on the WHO recommendations. Malaria risk factors will be analyzed using logistic regression. Multilevel logistic regression will be applied to estimate the risk score for malaria.

**Results:**

Of the total participants, 99.46% (1495/1503) of rural adults from 49 villages in the ENTP participated in a face-to-face interview from October to December 2019. The study results are expected to be published in peer-reviewed journals.

**Conclusions:**

The best malaria risk prediction model will be developed in this study. In this protocol, we developed a methodology to provide new evidence to guide health policy in supporting the ENTP government’s expectation to achieve the malaria-free rating by 2030.

**International Registered Report Identifier (IRRID):**

DERR1-10.2196/23545

## Introduction

### Background

Malaria is a major global health problem, with an estimated 3.9 billion people living at risk of malaria infection [1]. In 2018, the World Health Organization (WHO) reported 228 million cases, 3.5% of which were from Southeast Asian (SEA) countries [1]. The action plan of the region indicates that all countries in the region will be malaria-free zones by 2030 [2]. Two countries, the Maldives and Sri Lanka, have been certified malaria-free areas by the WHO, whereas India and Indonesia are still affected by malaria, contributing 85% and 13% to the total number of malaria cases in the region, respectively [1].

Indonesia is a SEA country, with a total population of approximately 265 million [3]. It has a diverse ethnic composition, with 1340 ethnic groups distributed from Sabang to Merauke [4]. A significant reduction in the transmission of malaria in various provinces in Indonesia has occurred since the country has implemented its national commitment to eliminate malaria. As a result of this commitment, 285 of 514 districts (55.5%) achieved malaria elimination in 2018 [5]. However, none of the districts in Papua, West Papua, Maluku, North Maluku, and the East Nusa Tenggara Province (ENTP), Indonesia, have met the malaria elimination area but are committed to elimination by 2030 [5].

The WHO stated that the entire Indonesian population is at risk of contracting malaria and approximately 6.4% of this population have a high risk [1]. The annual parasite incidence (API) survey in 2018 reported that the national API value was 0.84 per 1000 people and that it varied across the 34 provinces [5]. The highest API value was found in Papua province at 52.99 per 1000 people, and in the ENTP (the focus of the proposed study), the API is 3.42 per 1000 people [5]. Over the past decade, there has been a steady decrease in API at the national level in Indonesia from 1.8 per 1000 in 2009 to 0.84 per 1000 in 2018, with this trend observed in most provinces. Despite the consistent decrease in the API value in the ENTP from 13.7 per 1000 people in 2014 [6] to 3.42 per 1000 people in 2018 [5], the API value is well above the national API. As epidemiological malaria research as well as the knowledge of the significant value of API in the ENTP are limited, this study focuses on malaria in the ENTP.

### Problem Statement and Justification

This study aims to address existing gaps in data focusing on knowledge, attitude, and practice (KAP) toward aspects of malaria; access to and use of long-lasting insecticide-treated nets (LLINs); and malaria risk factors in the ENTP. Several KAP studies on malaria have been conducted in Indonesia [7-9]. However, most of these studies were conducted in western Indonesia, a categorized malaria-free zone, and most were directed at the subdistrict and village levels. One population-based study of 4050 respondents in North Maluku province indicated that although 93.6% of the population realized that malaria is a dangerous disease, almost all respondents (98%) did not know the main causes of malaria [8]. However, 30% of the respondents in the study were children between the ages of 5 and 9 years, making them unsuitable candidates to measure the level of knowledge of a particular community. Another study focused on the KAP toward aspects of malaria at the province level in Central Java provinces [9]. However, the practice of communities using LLINs was not investigated in that study. Studies in various settings have shown that the practice of communities sleeping under LLINs has reduced the transmission of malaria [10,11], and the WHO has recommended using LLINs as the best method to prevent malaria [12].

The increased coverage of LLINs is a key intervention strategy to reduce malaria in Indonesia [13]. From August to October 2017, the Indonesian government implemented a malaria control acceleration program through the mass distribution of LLINs in 67 districts in 5 provinces in the eastern part of Indonesia (Papua, West Papua, Maluku, North Maluku, and ENTP), and of the 22 districts in the ENTP, 15 received the acceleration program [14]. However, despite a 76% increase in the distribution of LLINs from 2015 to 2017, there has been limited publication about access to and coverage of LLINs at the community level in Indonesia [15]. The universal coverage of LLINs [16] has not yet been investigated in the ENTP. A better understanding of the coverage of these indicators would play an important role in developing strategies for a stronger malaria control program in a country [17].

Several epidemiological studies have been conducted to understand the etiology in Indonesia as part of the global effort to eliminate malaria in the country [18-26]. Studies on the social and demographic aspects of malaria have been conducted in Papua province [18,19], Aceh province [26], Maluku province [20], and North Maluku province [21]. However, the effect of the use of LLINs on malaria infection in rural communities was not investigated in any of these studies. Several studies investigated the risk factors for malaria in the ENTP [27,28]. However, either the sample sizes of those studies were too small or the studies were conducted at the subdistrict and village levels. Moreover, although some studies have been conducted at the population level in the ENTP [22-25], they did not evaluate the impact of malaria knowledge, ethnic variations, and coverage of LLINs on the transmission of malaria. Examining the determinant factors of malaria more comprehensively would provide a better understanding of malaria epidemiology and enable experts to identify the important predictors of malaria risk in various environmental settings [29]. A recent study showed that factors associated with self-reported malaria varied between provinces, indicating that the local determinants of malaria risk factors existed at the individual, household, and community levels [20]. Therefore, this cross-sectional study will fill these gaps with the following objectives:

1. Determining awareness and KAP among adults toward various aspects of malaria and their associated factors, including sociodemographic and ethnic groups.
2. Assessing the gap between coverage of and access to and use of LLINs within households.
3. Estimating the prevalence of and factors associated with malaria in rural adults in the ENTP.
4. Developing a risk prediction model for malaria to tailor appropriate interventions.

This study is expected to provide significant findings to comprehensively explain the epidemiology of malaria in the ENTP. The gaps in the knowledge of malaria, the practices of communities using core prevention methods such as LLINs, the practices of malaria treatment–seeking behavior of communities of various ethnicities, and the main malaria risk factors will be identified. These results will help public health policy makers in Indonesia to develop local context-based malaria policies as part of the global effort to achieve a malaria-free zone in Indonesia by 2030. This model can then be implemented in similar socioeconomic settings in other countries.

## Methods

### Study Population

**Figure 1:**
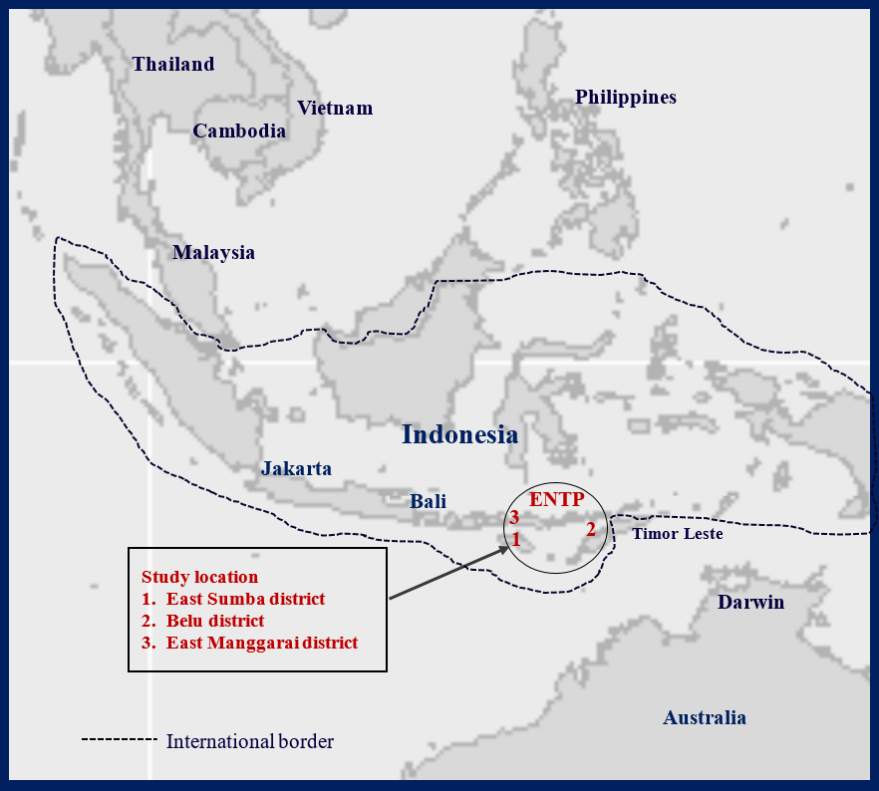
Map of study sites. ENTP: East Nusa Tenggara Province.

The ENTP, located in the eastern part of Indonesia, is one of the 34 provinces in the country. The total population is approximately 5.3 million, comprising 2.6 million males and 2.7 million females [30]. This project was conducted in 3 districts—East Sumba, Belu, and East Manggarai—based on the API values of malaria in the region. The East Sumba district has an estimated number of households of about 52,176 [30] and the highest API [5]. The East Manggarai district has an estimated number of households of approximately 55,372 [30] and has the lowest API [5]. Belu district has an estimated number of households of about 46,865 [30] and a moderate API [5]. The 3 districts are shown in Figure 1.

### Sample Size and Statistical Power

A cross-sectional study was conducted. The base sample size (n) was calculated using the following formula for a dichotomous outcome in the prevalence study [31]:

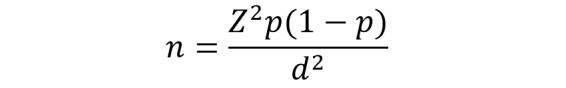



where p is the prevalence of malaria in ENTP=1.99% [32], Z is the confidence level at 95% (standard value of 1.96), d (relative precision)=0.01125, therefore:

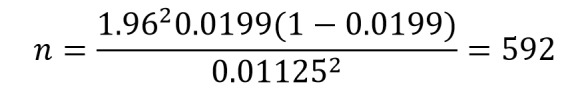



Considering an intraclass correlation coefficient (=0.04) to study malaria prevention methods in Indonesia [33] and a cluster size of n=30 adults per village, the design effect (DEFF) was defined as described by the WHO [34]:

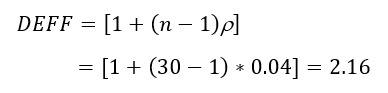



The adjusted sample size 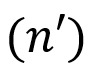 after considering DEFF is as follows:

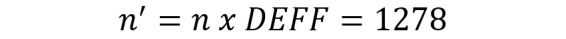



Finally, considering an 85% participation rate (y), the required sample size 
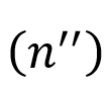
 was calculated, as defined by the WHO [34]:

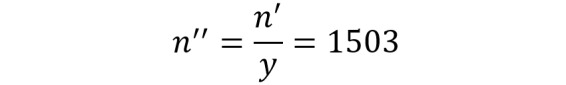



### Sampling Frame

**Figure 2:**
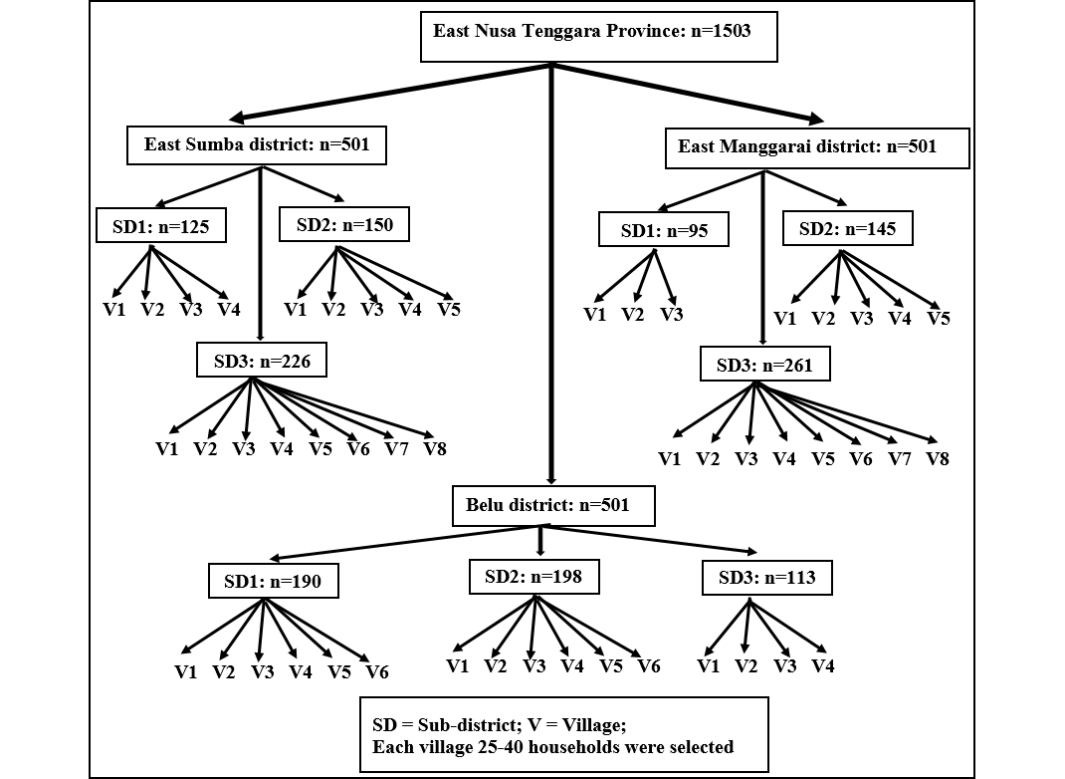
Flow chart for selecting clusters and households in ENTP Indonesia. ENTP: East Nusa Tenggara Province.

A multistage cluster sampling procedure with a systematic random sampling procedure at cluster level 4 was applied for this cross-sectional study. First, out of the 22 districts in the province, 3 were selected based on the malaria API. Second, in each selected district, 3 subdistricts were randomly selected. The number of clusters or villages was selected from each subdistrict based on their relative population sizes. Finally, for each village, a systematic random sampling technique was applied to interview 1 adult per household. Data collection was conducted in 49 villages, with 20 to 40 participants per village proportionate to the population sizes of the villages, as shown in Figure 2.

### Recruitment Strategy

First, a cover letter from the chief investigator was sent to the Governor of ENTP to seek approval. Second, the project team sought approval from the head of the East Sumba, Belu, and East Manggarai districts. Once the approval letters at district levels had been received, the research team approached the heads of the subdistricts for their approval. After the research team received written approvals from subdistricts and village leaders, the interviewer started approaching the prospective participants. In the selected households, the research team first approached the heads of households for interviews. In case the household heads, either husband or wife, were absent, any residents above 18 years of age could serve as study participants [35]. As we wanted to investigate malaria knowledge of adults in the ENTP, any potential participants less than 18 years of age were excluded from the study.

### Quality Assurance

Data collectors, with a health education background, participated in a 1-day intensive training session before commencing household surveys. Intensive training was conducted in Borong, Belu, and Waingapu cities. The main objective of the training is to improve data collectors’ knowledge of the various aspects of malaria and improve their understanding of the importance of strict adherence to the sampling protocol. Through this intensive training, the interview process will run smoothly and reduce the potential coercion of the participants.

### Questionnaires

A structured questionnaire was modified from a validated questionnaire [36,37]. Overall, there were 6 main parts of the questionnaire. The first section discusses the demographic information of the participants. We collected information on gender, age, education level, occupation, family size, household income, income of the household head, the main material of the house, ownership of durable assets, access to drinking water, those nearest to the health facilities, and distance to the nearest health facilities. The second part of the questionnaire focused on the general knowledge of malaria. In this part, we asked participants about malaria knowledge, including symptoms of, main causes of, and preventative action to prevent malaria. Next, we collected information on the treatment-seeking behavior of malaria. In this part, we collected information on when and where participants would find the treatment if they or their family members are affected by malaria. Personal protection practices of participants were collected in the fourth section of the questionnaire. In the fifth section, we collected information on self-reported malaria of participants and how they treated their malaria. Finally, at the end of the questionnaire, we obtained data on the demographic information of family members, particularly for those with children aged below 5 years. The practice of sleeping under bed nets for children was also documented. The comprehensive questions for each section are presented in the questionnaire, as shown in Multimedia Appendix 1.

### Outcome Variables

The outcome variables of the study will be divided into 10 themes. The first outcome variable is the malaria awareness index. This index will be measured by 10 questions related to malaria knowledge on symptoms of, main causes of, prevention actions for, and treatment-seeking behavior for malaria. In each question, 1 mark will be awarded for each correct answer, whereas incorrect answers will be given no points. The total marks for the 10 questions will be evaluated as follows. The level of knowledge with an accurate rate above 80%, 60% to 79%, 1% to 59%, and 0% will be classified as excellent, good, poor, and zero level of awareness, respectively. The rank of excellent and good will be categorized as having malaria awareness, whereas poor and zero levels of awareness will be classified as unaware of malaria [38,39].

The second outcome variable is the predictor of malaria awareness. Malaria awareness of participants will be tabulated based on sociodemographic and environmental factors.

The third outcome variable is the predictor of misconception of the main symptoms and transmission mode of malaria. The main symptom of malaria is fever [40]. All respondents who mentioned other symptoms for the main symptoms of malaria will be classified as misconception. The transmission mode of malaria is mosquito bites [41,42]. All respondents stating other transmission modes of malaria will be classified as misconception. The misconception about the main symptoms and transmission mode of malaria for all participants will be tabulated based on their sociodemographic and environmental factors.

The fourth outcome variable is the gap between knowledge and practice of malaria prevention measures. Knowledge and practice of various malaria prevention measures will be identified for each respondent. The gap between knowledge and practice will be calculated. Finally, the gap between knowledge and practice will be tabulated based on their sociodemographic and environmental factors.

The fifth outcome variable is the malaria treatment–seeking behavior of participants. Appropriate malaria treatment–seeking behavior (AMTSB) is defined as seeking treatment from professional health centers or at health facilities within 24 hours of symptom onset [43]. All respondents stating that they will visit professional health centers within 24 hours were classified as having AMTSB. The AMTSB of participants will be tabulated based on their sociodemographic and environmental factors. Next, the poor understanding of AMTSB is defined as seeking treatment after 24 hours or at nonhealth facilities [44]. All respondents stating that they will visit professional health centers beyond 24 hours or seek treatment at nonhealth facilities will be classified as having a poor understanding of AMTSB. The poor understanding of AMTSB will be tabulated based on their sociodemographic and environmental factors.

The sixth outcome variable is the predictor of bed net ownership and usage among adults. Bed net ownership will be identified for each respondent. The types of bed nets owned are LLINs, non-LLINs, or both of them. This ownership will be classified for each respondent. The use of bed nets and the type of bed net used the previous night (sleeping under the bet net on the previous night before the interview) will be identified for each respondent. Finally, bed net ownership and usage will be tabulated based on their sociodemographic and environmental factors.

The seventh outcome variable is the coverage of LLINs, universal access to LLINs, and the used gap of LLINs. Following the method recommended by the WHO [12,17], 6 indicators will be investigated. They are the proportion of households with at least one LLIN (P1); the proportion of households with at least one usable LLIN (P2); the proportion of households with access to LLINs (P3); the proportion of households with access to LLINs if any LLINs are present (P4); the proportion of households using LLINs the previous night (P5); and the proportion of households that used LLINs the previous night if accessed (P6). Finally, the access gap will be defined as 1-P3, and the use gap will be defined as 1-P6 [17].

The eighth outcome variables are bed net usage and associated factors among children aged below 5 years in the ENTP. The proportion of children aged below 5 years who slept under an LLIN the previous night (P7) is one of the important indicators recommended by the WHO [16]. The use of bed nets and the type of bed net used the previous night (LLINs and non-LLINs) will be identified for each respondent having children aged below 5 years. P7 will be tabulated based on the sociodemographic and environmental factors of the parents.

The ninth outcome variable is malaria prevalence and its associated factors. The prevalence of malaria based on self-reported malaria among respondents will be reported. An adult in a household was asked whether they had been diagnosed with positive laboratory-confirmed malaria by local health providers or physicians in the past 12 months. The accuracy of the response was validated by asking the respondent a supplementary question about the symptoms of their malaria events. Approximately 20% of the responses were also validated by contacting their health care providers.

The 10th outcome variable is the prediction of the village with the highest risk of malaria. All villages will be ranked based on the malaria awareness of the community, level of misconception of main symptoms and transmission mode of malaria, gap between knowledge and practice of malaria prevention, level of treatment-seeking behavior, and universal access to bed nets.

### Statistical Analysis

For the first outcome, participants’ sociodemographic characteristics, including gender, age group, education level, and socioeconomic status (SES), will be reported using descriptive statistics. A chi-square test will be applied to evaluate the association of basic malaria understanding, basic malaria knowledge, the level of malaria knowledge, and the level of malaria awareness among 3 types of malaria endemic settings (MESs). A *P* value <.05 will be considered statistically significant. SPSS version 27 (IBM Corporation) will be used for analyses.

For the second outcome, participants’ sociodemographic and environmental characteristics, including gender, age group, education level, ethnicity, SES, family size, health facilities close to their house, and distance to the nearest health facilities will be reported using descriptive statistics. A chi-square test will be applied to evaluate the association between malaria awareness and the sociodemographic and environmental characteristics of respondents. Bivariate logistic regression will be performed to explore the association between predictors and malaria awareness of participants. Potential explanatory predictors available with statistically significant differences (*P*<.05) will finally be retained in this model.

For the third outcome, sociodemographic and environmental characteristics of participants hearing malaria terms, including gender, age group, education level, ethnicity, SES, family size, health facilities close to their house, and distance to the nearest health facilities, will be reported using descriptive statistics. Perception of participants on malaria symptoms, main symptoms, transmission mode of malaria, misconception of main symptoms, and transmission mode of malaria will be reported in percentage. A chi-square test will be applied to evaluate the association between the misconception and sociodemographic and environmental characteristics of respondents. Bivariate logistic regression will be performed to explore the association between the predictors and the misconception of participants. Potential explanatory predictors available with statistically significant differences (*P*<.05) will finally be retained in this model.

For the fourth outcome, sociodemographic and environmental characteristics of participants hearing malaria terms, including gender, age group, education level, ethnicity, SES, family size, health facilities close to their house, and distance to the nearest health facilities, will be reported using descriptive statistics. The perception of participants on knowledge and practice of the 13 malaria prevention methods will be presented in the proportion. The gap between knowledge and practice in the 13 malaria prevention methods will be presented in the proportion. The level of malaria prevention measures knowledge and practice of sleeping under any bed net to prevent malaria will be tabulated by different sociodemographic and environmental characteristics. A chi-square test will be applied to evaluate the association between knowledge and practice of sleeping under any bed net to prevent malaria and the sociodemographic and environmental characteristics of respondents. Bivariate logistic regression will be performed to explore the association between predictors and knowledge and practice of sleeping under any bed net to prevent malaria. Potential explanatory predictors available with statistically significant differences (*P*<.05) will finally remain in this model. A chi-square test will be applied to evaluate the association between knowledge and practice of sleeping under LLINs to prevent malaria and the sociodemographic and environmental characteristics of respondents. Bivariate logistic regression will be performed to explore the association between predictors and knowledge and practice of sleeping under LLINs to prevent malaria. Potential explanatory predictors available with statistically significant differences (*P*<.05) will finally be retained in this model.

For the fifth outcome, the sociodemographic and environmental characteristics of participants, including gender, age group, education level, ethnicity, SES, family size, health facilities close to their house, and distance to the nearest health facilities, will be reported using descriptive statistics. Perception of finding treatment if respondents or their family members have any symptoms of malaria will be presented in the proportion. The proportion of respondents seeking malaria treatment after 24 hours and at nonhealth facilities and having a poor understanding of AMTSB will be tabulated by sociodemographic and environmental characteristics of participants. Bivariate logistic regression will be performed to explore the association between predictors and the perception of respondents on seeking malaria treatment after 24 hours and at nonhealth facilities and having a poor understanding of AMTSB. Potential explanatory predictors available with statistically significant differences (*P*<.05) will finally be retained in this model.

For the sixth outcome, the sociodemographic and environmental characteristics of participants, including gender, age group, education level, ethnicity, SES, family size, health facilities close to their house, and distance to the nearest health facilities, will be reported using descriptive statistics. The ownership and use of the bed net of participants will be presented in the proportion. The ownership of any bed net, LLINs, and non-LLINs will be tabulated by the different sociodemographic and environmental characteristics of respondents. Bivariate logistic regression will be performed to explore the association between predictors and the ownership of any bed net, LLINs, and non-LLINs. The use of any bed net, LLINs, and non-LLINs will be tabulated by different sociodemographic and environmental characteristics of respondents. Bivariate logistic regression will be performed to explore the association between predictors and the use of any bed net, LLINs, and non-LLINs. Potential explanatory predictors available with statistically significant differences (*P*<.05) will finally be retained in this model.

For the seventh outcome, the sociodemographic and environmental characteristics of participants, including gender, age group, education level, ethnicity, SES, family size, health facilities close to their house, and distance to the nearest health facilities, will be reported using descriptive statistics. Applying the method provided by the Roll Back Malaria Monitoring and Evaluation Reference Group [16], the gap between the coverage of and access to and use of LLINs will be evaluated. First, the 6 main indicators will be calculated.

The first indicator is the proportion of households with at least one LLIN (P1). The numerator consists of all households with at least one (or 2) LLINs, and the denominator is the total number of sampled households. The second indicator is the proportion of households with at least one usable LLIN (P2). The numerator consists of all households with at least one usable (no visible rift on the net) LLIN, and the denominator is the total number of sampled households.

The third indicator is the proportion of households with access to LLINs (P3). This indicator is defined as households with at least one LLIN for every 2 people and is called *sufficient access*. The numerator contains all households with at least one LLIN for every 2 people, whereas the denominator is the total number of sampled households. The fourth indicator is the proportion of households with access to LLINs, if any LLINs are present (P4). The numerator contains all households with at least one LLIN for every 2 people, whereas the denominator is the total number of sampled households with at least one LLIN.

The fifth indicator is the proportion of households using LLINs the previous night (P5). The numerator contains all households whose members slept under LLINs the previous night, and the denominator is the total number of households in the sample. The sixth indicator is the proportion of households that used LLINs the previous night if accessed (P6). The numerator contains all households whose members slept under LLINs the previous night, and the denominator is the total number of households with sufficient access to LLINs.

The coverage indicators will be investigated by P1 and P2. The access indicators will be identified by P3 and P4. The use indicators will be investigated by P5 and P6. Households not having at least one LLIN for every 2 people (1-P3) are defined as having insufficient access to LLINs or having *an access gap*. Households that did not have access to LLINs despite possessing LLINs, which is 1-P4, are defined as intrahousehold net gaps. A household that did not use LLINs the previous night despite having access, which is 1-P6, is defined as *the used gap of LLINs.*

Descriptive analysis will be conducted to show the distribution of different characteristics of the respondents based on these 6 indicators. To evaluate the association between sociodemographic and environmental characteristics and LLINs’ coverage, access, and use, the chi-square test will be applied.

For the eighth outcome, the sociodemographic and environmental characteristics of participants, including gender, age group, education level, ethnicity, SES, family size, health facilities close to their house, and distance to the nearest health facilities, will be reported using descriptive statistics. The use of bed nets (P7a), LLINs (P7b), and non-LLINs (P7c) by children aged below 5 years will be calculated in the form of proportion. P7a, P7b, and P7c will be tabulated based on the sociodemographic and environmental factors of the parents. Bivariate logistic regression will be performed to explore the association between predictors and the use of any bed net, LLINs, and non-LLINs by children aged below 5 years. Potential explanatory predictors available with statistically significant differences (*P*<.05) will finally be retained in this model.

For the ninth outcome, the sociodemographic and environmental characteristics of participants, including gender, age group, education level, ethnicity, SES, family size, health facilities close to their house, and distance to the nearest health facilities, will be reported using descriptive statistics. Self-reported malaria of participants will be tabulated based on the 4 aspects of malaria, including malaria awareness, level of malaria knowledge, level of misconception of main symptoms and transmission mode of malaria, knowledge and practice of malaria prevention methods, level of treatment-seeking behavior, bed net ownership and usage, and sufficient access to LLINs. All associations between covariates and self-reported malaria will first be examined using bivariate logistic regression. All significant variables with *P* values of Wald test ≤.01 will be considered statistically significant and will be included in the final multivariable logistic regression. The risk prediction model for malaria will be developed based on the significant factors associated with the prevalence of malaria. The prediction value will be assigned based on the severity of the factors, either with binary or multicategory factors.

For the 10th outcome, a risk prediction model for malaria will be developed based on the significant factors associated with the prevalence of malaria. Prediction values will be assigned based on the severity of the factors by applying multilevel modeling. The village variables will be approximated by aggregating individual variables at the village level. The village-level variables will be the location of the village, average distance to health facilities, accessibility to the village, coverage of LLINs, level of malaria knowledge, average education level of the community, and wealth quintile. The logistic regression framework will be applied for modelling the unadjusted, adjusted, and final analyses. Once the best model is identified, a malaria risk scoring system will be developed and the allocation of points for each variable will be based on the magnitude of its regression coefficients [45]. The sum of points for each variable will be used as an approximation to rank the village from the highest to the lowest risk of malaria in the future [45].

### Ethics and Dissemination

The Declaration of Helsinki was adhered to ensure that the rights, integrity, and confidentiality of the respondents are strictly protected. All respondents signed consent forms before being interviewed. For this study, we received human ethics approval from the Swinburne University of Technology Human Ethics Committee (Ethics ID: 20191428-1490) and from the Health Research Ethics Committee of the National Institute of Health Research and Development of the Indonesian Ministry of Health (Ethics ID: LB.02.01/2/KE.418/2019). The results of this cross-sectional study will be presented at international conferences and published in peer-reviewed journals.

## Results

The time frame of the project is presented in Table 1.

Primary data were collected from October to December 2019. From a total of participants, 99.46% (1495/1503) of rural adults from 49 villages in the ENTP participated in a face-to-face interview. The project is in progress to draft papers that will be published in peer-reviewed journals.

**Table 1:** Timeline of research phases.

Research phases	June-December 2018	January-December 2019	January-May 2020	June 2020-July 2022
Questionnaire and question guide development	✓			
Ethics approval		✓		
Training of field workers		✓		
Household surveys		✓		
Data entering			✓	
Data analysis				✓
Manuscript preparation and submission				✓

## Discussion

### Principal Findings

Our study endeavored to explore the contribution of differential risk factors for malaria in rural ENTP. We consider KAP as an important societal risk factor given that contemporary studies in South Asia revealed that low levels of general knowledge on transmission and prevention of malaria in the community in the region had been identified both in the general public and community health practitioners [46]. A study in Myanmar [47] and Nepal [48] highlighted that there is a misconception in the malaria transmission mode in the community and that poor malaria knowledge leads to poor health treatment–seeking behavior for malaria [47]. This study will provide a unique opportunity to identify the gaps in KAP of various aspects of malaria in rural communities of the ENTP. These findings may be published in prestigious journals allowing other malaria experts to compare and contrast the malaria KAP of communities from various settings, including from the ENTP.

This will be the first population-based study revealing KAP toward aspects of malaria in rural adults from 3 different MES in the ENTP. We allocated the balance sample from 3 different MES, allowing us to compare the characteristics of rural adults of their malaria knowledge, practice of malaria prevention method, and treatment-seeking behavior. Furthermore, the study participants selected were solely those living in rural areas, so the true characteristics of the rural population in various aspects of malaria would be revealed through this study. A good understanding of the malaria KAP of the rural population is critical considering that most malaria cases globally were from rural populations [15,49,50]. In the Indonesian context, more than half of the malaria cases were contributed by rural communities [51].

The instruments used to collect the data were modified from a validated questionnaire with some modifications to capture the ownership and use of LLINs both in adults and children aged below 5 years in the ENTP. The assessment of the gap between the coverage of and access to and use of LLINs in the ENTP will be conducted based on the standardized indicators recommended by the WHO [16], allowing the findings to be compared with other national and international studies. Furthermore, the universal access of LLINs and their impact on malaria prevalence in the region could be revealed throughout this study. These findings will provide the best reference for malaria control programs in the region to support the Indonesian government’s expectation of achieving a malaria-free rating by 2030.

Despite the several benefits of this study, there are some limitations. First, adults were asked about their medical histories in the last year, specifically whether they or their children aged below 5 years had contracted malaria. This may reflect recall bias. Next, adult self-reported data will be used to estimate malaria prevalence and investigate the practices of prevention activities and the health and treatment-seeking behavior of communities. This might lead to the introduction of courtesy bias. However, the instruments used to obtain the data applied validated and reliable questionnaires that have been applied in various settings. The instruments were then administered by experienced local health workers.

### Conclusions

This research will be expected to provide significant findings to comprehensively explain the epidemiology of malaria in the ENTP. The gap in malaria knowledge, practice of communities in using core prevention methods such as LLINs, practice of malaria treatment–seeking behavior of communities with various ethnicities, and the main malaria risk factors will be recognized. These results could help stakeholders in the region to develop malaria policy based on the local context to support the global effort to become a malaria-free zone by 2030.

## Appendix

Multimedia Appendix 1 The questionnaire for primary data collection.

Multimedia Appendix 2 Ethics Approval of Swinburne University of Technology.

Multimedia Appendix 3 Ethics Approval of Indonesia Health Ministry.

Multimedia Appendix 4 Original peer-review reports from the Swindburne University of Technology.

## Abbreviations

AMTSB: appropriate malaria treatment–seeking behavior
API: annual parasite incidence
DEFF: design effect
ENTP: East Nusa Tenggara Province
KAP: knowledge, attitude, and practice
LLIN: long-lasting insecticide-treated nets
MES: malaria endemic setting
SEA: Southeast Asia
SES: socioeconomic status
WHO: World Health Organization
