# Supplementary material for: Epidemiology of Malaria in East Nusa Tenggara Province in Indonesia: Protocol for a Cross-sectional Study
Source: JMIR Res Protoc. 2021 Apr 9;10(4):e23545. doi: 10.2196/23545 (PMC8075045; doi:10.2196/23545)
Supplement: Multimedia Appendix 2 [file resprot_v10i4e23545_app2.pdf]

Swinburne University of Technology Human Research Ethics Committee

Approval certificate

The ethics application for your project Trend analysis of malaria prevalence and developing a predictive model for its associated risk factorshas been approved.

Chief Investigator:A/Prof. Amirul Islam

Ref: 20191428-1490

Approved Duration:11/9/2019 to 11/9/2023

I refer to the ethical review of the above project protocol by Swinburne's Human Research Ethics Committee (SUHREC) or its sub-committees.

I am pleased to advise that, as submitted to date, the project may proceed in line with standard on-going ethics clearance conditions outlined below.

- The approved duration is as shown above unless an extension request is subsequently approved.
- All human research activity undertaken under Swinburne auspices must conform to Swinburne and external regulatory standards, including the *National Statement on Ethical Conduct in Human Research (2018)* and with respect to secure data use, retention and disposal.
- The named Swinburne Chief Investigator/Supervisor remains responsible for any personnel appointed to or associated with the project being made aware of ethics clearance conditions, including research and consent procedures or instruments approved. Any change in Chief Investigator/Supervisor, and addition or removal of other personnel/students from the project, requires timely notification and SUHREC endorsement.
- The above project has been approved as submitted for ethical review by or on behalf of SUHREC. Amendments to approved procedures or instruments ordinarily require prior ethical appraisal/clearance from SUHREC for approval. SUHREC must be notified immediately or as soon as possible thereafter of (a) any serious or unexpected adverse effects on participants and any redress measures; (b) proposed changes in protocols; and (c) unforeseen events which might affect continued ethical acceptability of the project.
- At a minimum, an annual report on the progress of the project is required as well as at the conclusion (or abandonment) of the project.
- A duly authorised external or internal audit of the project may be undertaken at any time.
- Please forward this approval certificate to relevant members of the project team.

The following investigators have been approved to work on the project:

Chief Investigator

Amirul Islam

Associate Investigators

Steve Quinn

Student Investigators

Robertus Dole Guntur

Please contact the Swinburne Research Ethics Office if you have any queries.

Regards,

Dr Astrid Nordmann

on behalf of

Research Ethics Office

Swinburne University of Technology

P: +61 3 9214 3845 | E: resethics@swin.edu.au
