# Supplementary material for: Epidemiology of Malaria in East Nusa Tenggara Province in Indonesia: Protocol for a Cross-sectional Study
Source: JMIR Res Protoc. 2021 Apr 9;10(4):e23545. doi: 10.2196/23545 (PMC8075045; doi:10.2196/23545)
Supplement: Multimedia Appendix 3 [file resprot_v10i4e23545_app3.pdf]

KEMENTERIAN KESEHATAN REPUBLIK INDONESIA
BADAN PENELITIAN DAN PENGEMBANGAN KESEHATAN
Jalan Percetakan Negara No. 29 Jakarta 10560 Kotak Pos 1226
Telepon (021) 4261088 faksimile (021) 4243933
Laman : www.litbang.depkes.go.id Surat Elektronik :sesban@litbang.depkes.go.id

GERMAS

PERSETUJUAN ETIK
ETHICAL APPROVAL

No. : LB.02.01/2/KE.418/2019

Komisi Etik Penelitian Kesehatan, Badan Penelitian dan Pengembangan Kesehatan (KEPK-BPPK) dengan berdasarkan Deklarasi Helsinki, telah melakukan telaah, pembahasan dan penilaian melalui proses **Expedited.**
memutuskan protokol penelitian yang berjudul :

Health Research Ethics Committee, National Institute of Health Research and Development (HREC-NIHRD), in accordance with Helsinki Declaration, has conducted a thorough Expedited review of research protocol entitled :

"Trend Analysis of Malaria Prevalence and Developing a Predictive Model for Its Associated Risk Factors"

yang akan mengikutsertakan manusia sebagai partisipan/subyek penelitian; dengan Koordinator Penelitian :
in which will involve human participant(s). As Research Coordinator :

Robertus Dole Guntur

dapat diberikan **persetujuan etik**. Masa berlaku surat persetujuan etik ini adalah :
*has hereby declared the protocol is **approved** for implementation. This letter is valid from/to :*

11 November 2019 s/d **10 November 2020**

Jika ada perubahan protokol (amandemen) dan/atau perpanjangan penelitian, Ketua Pelaksana/Peneliti Utama harus mengajukan kembali protokol versi terbaru untuk kaji etik penelitian. Pada akhir penelitian, laporan pelaksanaan penelitian juga harus diserahkan kepada KEPK-BPPK.
Should there be any modification (amendment) and/or extention of the study, the Principal Investigator is required to resubmit the latest version of protocol for approval. The final summary reports should also be submitted to HREC-NIHRD.

Jakarta, 11 November 2019

Chair of HREC-NIHRD :

Ketua
Komisi Etik Penelitian Kesehatan
Badan Litbang Kesehatan,

Prof. Dr. M. Sudomo
