# Supplementary material for: Epidemiology of Malaria in East Nusa Tenggara Province in Indonesia: Protocol for a Cross-sectional Study
Source: JMIR Res Protoc. 2021 Apr 9;10(4):e23545. doi: 10.2196/23545 (PMC8075045; doi:10.2196/23545)
Supplement: Multimedia Appendix 4 [file resprot_v10i4e23545_app4.pdf]

Graduate Studies

CONFIRMATION OF CANDIDATURE REVIEW FORM for Doctoral and Masters Candidates

IMPORTANT INFORMATION AND INSTRUCTIONS

This form is for the assessment of the confirmation of candidature requirements for candidates enrolled in PhD, Professional Doctorate or Masters by Research programs. As per the guidelines in the Research Training Statement of Practice, all panel members must be in attendance at the meeting (either in-person or by phone/Skype); while all supervisors are encouraged to attend, the Principal Supervisor's attendance is required.

If this is a follow-up review, the candidate and all members of the Review Panel and Supervisory Team should review the outcomes of the initial Confirmation of Candidature meeting.

At least **10 business days** prior to the review meeting, candidates must submit a written research plan as per the guidelines in the Research Training Statement of Practice.

INSTRUCTIONS FOR THE PANEL CHAIR & PRINCIPAL SUPERVISOR

Meeting Structure

A total of 90 minutes should be allocated for the Confirmation of Candidature Review meeting. A suggested format for the review is as follows:

1) Candidate gives a presentation – 15 minutes
2) Discussion among the panellists and candidate – 35 minutes
3) Candidate leaves the room. Supervisors are given an opportunity to discuss with the panel the candidate's progress and any issues – 10 minutes
4) Supervisors leave the room. Candidate is given an opportunity to discuss with the panel their progress and any issues – 10 minutes
5) Panellists confer on their own, without the candidate or supervisors present – 10 minutes
6) Supervisors return to the room. Candidate receives feedback and the proposed recommendation that the panel will make to the Faculty. Candidate is advised of next steps in relation to the progress of their research. – 10 minutes.

Submitting the review report for Faculty review/approval

7) The panel chair is responsible for completing this review report electronically (handwritten reports will not be accepted) with input from the panel. A final draft of the report may be shared with the supervisory team but is not final until approved by the Faculty.
8) The panel chair must send the report to HDRCandidature@swin.edu.au **within 10 business days** of the review meeting.
9) Once approved by the Faculty, Graduate Studies will send the final copy of the report to the supervisors and candidate. The Principal Supervisor is responsible for discussing the report with their candidate.

More information can be found in the Research Training Statement of Practice:
http://www.swinburne.edu.au/intranet/research/swinburne-research/research-governance--policy/

Correspondence
Please note that all correspondence will be sent to your Swinburne email account (name@swin.edu.au).

Privacy
This form will be kept on your student record in accordance with University Privacy Policy, which can be viewed at https://www.swinburne.edu.au/privacy/.

Graduate Studies

CONFIRMATION OF CANDIDATURE REVIEW FORM for Doctoral and Masters Candidates

SECTION A: CANDIDATE DETAILS

Student ID: 101750704 Date: 12/07/2019

☑ This is the first review
☐ This is a follow-up review

Surname: Guntur Given Names: Robertus Dole

Faculty of Enrolment: Health, Arts and Design

School / Centre / Department: Statistics, Data Science and Epidemiology

Program: PhD by Publication

Study Load: ☑ Full Time ☐ Part Time Study Location: ☐ Australia ☐ Overseas

Sponsoring/Partner Organisation (if applicable): Department of Foreign Affairs and Trade: Australia Awards Scholarship

SUPERVISORY TEAM:

Name of Principal Supervisor: Amirul Islam

Co-Supervisor: Steven Quinn

Co-Supervisor/Associate: (if applicable)

Other supervisor(s): (if applicable)

CONDITIONS

For candidates with conditions on their offer of candidature letter, only: Have the conditions been met?

☐ **Yes** ☐ **No**

COURSEWORK REQUIREMENTS

For candidates enrolled in the (GC-RESIM), have the two core units been completed? ☑ Yes ☐ No

If no, what units are remaining and what is the timeline for completion?

For candidates enrolled in any other course units, have the appropriate units been completed? ☐ Yes ☑ No

If no, what units are remaining and what is the timeline for completion?

MFP60001 and MFP60002 to be completed by June 2021

CONFIRMATION OF CANDIDATURE REVIEW FORM
for Doctoral and Masters Candidates

ETHICS

Does the project require Swinburne ethics approval? ☑ Yes ☐ No

If Yes, has Swinburne ethics approval been obtained? ☐ Yes ☑ No

If Yes, Ethics ID Number:

If No, please specify the expected date by which the ethics application will be submitted:

Are any other ethics approvals required for this project (e.g., from an external organisation)?

☐ Yes ☑ No If yes, please explain:

SECTION B: KEY DATES AND REVIEW DEADLINES

Please complete this section noting past reviews, this review and expected submission date.

Candidature Commencement Date

**Review**	**Months from commencement date:** (unless an extension has been approved or leave period has changed review deadlines):	**Date Scheduled/Held**
Pre-Confirmation of Candidature Review:	Date passed	
Confirmation of Candidature Review:	For PhD, 9-12 months FTE<br>For Masters, 6-9 months FTE	12th July 2019
Follow-up review (if needed):	Within 3 months of the initial review	
Mid-Candidature Review:	For PhD only, 21-24 months FTE	
Draft Thesis Review:	For PhD, 33-36 months FTE<br>For Masters, 18-21 months FTE	
Submission:	For PhD, 36 months FTE<br>For Masters, 24 months FTE	

SECTION C: APPROVED PANEL MEMBERS

(Note: panel membership should remain the same for all progress reviews; where a change is needed, a new panel nomination form must be submitted at least one month ahead of the proposed review meeting date)

**Panel Chair**	Denny Meyer
**Panel Member 2**	Abdullah Al Mahmud
**Panel Member 3**	Pragalathan Apputhurai
**Panel Member 4** (optional)	
**Procedural Chair (ADRT)** (if advised by ADRD)	

Graduate Studies

CONFIRMATION OF CANDIDATURE REVIEW FORM
for Doctoral and Masters Candidates

SECTION D: ASSESSING PERFORMANCE

This section is to be filled out by the Panel (or Procedural) Chair during the review meeting. Please select either 'yes' or 'no' or 'needs improvement' to the following criteria and provide specific written feedback, where relevant.

A) Has the candidate provided a research proposal that is of sufficient quality according to disciplinary expectations?

☑ Yes ☐ Yes, but needs improvement ☐ No

Very good

B) Was the quality of the candidate's presentation appropriate per disciplinary expectations (e.g., length; presentation skills; content)?

☑ Yes ☐ Yes, but needs improvement ☐ No

Excellent

C) Has the candidate completed the work required within the expected timeframe for their program (including the two core units of the Graduate Certificate of Research and Innovation Management where applicable)?

☑ Yes ☐ No

D) Has the candidate shown initiative and intellectual engagement consistent with the requirements of the research program and level of study?

☑ Yes ☐ Yes, but needs improvement ☐ No

E) Has the candidate conducted their research with integrity?

☑ Yes ☐ Yes, but needs improvement ☐ No

CONFIRMATION OF CANDIDATURE REVIEW FORM for Doctoral and Masters Candidates

F) Is the timeline for completion provided by the candidate in their written report (including the completion of any remaining units for the Graduate Certificate of Research and Innovation Management, where applicable) reasonable for a timely completion?

☐ Yes ☑ Yes, but needs improvement ☐ No

There is a four year scholarship for this project but Swinburne requires a plan for only three years. The plan needs to be modified accordingly and advice should be sought by the principal supervisor to ensure that the sscholarship is not jeopardised.

G) Is the project feasible in terms of resources, budget, access to data collection sites, etc.?

☐ Yes ☑ Yes, but needs improvement ☐ No

There is some concern that the budget will not be sufficient to cover the primary data collection. The help of friends of the student in the dta collection process needs to be formalised.

H) Has the candidate provided a reference list?

☑ Yes ☐ No

I) Has the candidate provided a plan for disseminating the research, including a list of any publications, awards, presentations or exhibitions that have been produced to date?

☐ Yes ☑ Yes, but needs improvement ☐ No

The Government of Indonesia is supportive of this project. Ideally this support should be fomalised and plans should be made to formally communicate the results of the research to the Indonesian Government. This will ensure that the results guide the National Malaria Control Program of the Indonesian Government.

J) If a doctoral candidate, will the research lead to a significant academic contribution to the discipline?

☑ Yes ☐ No

K) Has the candidate outlined the potential contribution that the research will make to the economy, society, environment or culture, beyond the contribution to academic research?

☑ Yes ☐ Yes, but needs improvement ☐ No

L) Overall, has the candidate displayed progress at a satisfactory level?

☑ Yes ☐ Yes, but needs improvement ☐ No

Were any concerns raised by the candidate? ☑ Yes ☐ No

The student would like more regular meeting with the co-supervisor. The student is also reporting a high level of stress.

Were any concerns raised by the supervisor(s)? ☑ Yes ☐ No

A more formal meeting schedule is recommended.

Does the candidate need language support? ☑ Yes ☐ No

A friend of the student ensured that the confirmation report was of a good standard. Language training is a better option for the future.

Additional feedback

The student is commended on very good progress. However, it is suggested that regular breaks are essential in order to maintain good mental health.
It is recommended that meetings be formalised, once a week with the primary supervisor and at least once a month with both supervisors. An agenda and minutes should ideally be prepared by the student for all meetings.
The student is reminded that a Bonferroni correction needs to be applied when multiple chi-squared tests of association are used to address the same hypothesis. In addition it is recommended that when the importance of various factors are being compared in a model for malaria prevalend, the nesting of the data (e.g. within countries or villages) should be considered, perhaps using multi-level modelling. Weightin of the results for each village should also be considered, in order to ensure that the final results are more representative of the research population.

CONFIRMATION OF CANDIDATURE REVIEW FORM for Doctoral and Masters Candidates

IF THIS IS THE INITIAL CONFIRMATION OF CANDIDATURE REVIEW, COMPLETE

- SECTION E (PANEL RECOMMENDATION)
- SECTION F (ACTION PLAN) IF APPLICABLE

IF THIS IS THE FOLLOW-UP INITIAL CONFIRMATION OF CANDIDATURE REVIEW, COMPLETE

- SECTION G (PANEL RECOMMENDATION)
- SECTIONS H-J (CONVERSION) IF APPLICABLE

SECTION E: CONFIRMATION OF CANDIDATURE PANEL RECOMMENDATION

Please indicate the Panel's recommendation:

☑ **Pass: Candidature is to be confirmed**

If a Masters candidate, is the candidate intending to convert to a PhD? ☐ Yes ☐ No

If Yes, does the panel support this recommendation? ☐ Yes ☐ No
Please fill in Section H of this form.

☐ **Not passed: candidate to receive a formal warning of unsatisfactory process OR (for iPhD students, only) the candidate will not progress to the PhD component of the Integrated PhD program.**

The project plan requires additional development, the candidate has not yet completed conditions listed on their candidature offer letter, the candidate has not completed the core units of the Graduate Certificate of Research and Innovation Management (where applicable), and/or there has been a lack of timely progress to this stage of candidature.

A candidate who receives a formal warning is required to develop, in consultation with the Principal Supervisor, an Action Plan (see Section F) **within 10 business days** of the Confirmation Review meeting. The Action Plan developed requires endorsement from the Review Panel Chair. The Principal Supervisor is required to advise the Review Panel Chair and HDRCandidature@swin.edu.au when the candidate has completed all elements of the approved Action Plan and is ready to undertake the follow-up presentation.

The candidate is also required to undertake a follow-up progress review **within three months** of the first Confirmation review. If satisfactory progress is not demonstrated at that time, the candidature may be terminated or, for doctoral students only, recommended for conversion to a Masters by Research.

Date by which Follow-up Review Meeting must be held:

Confirmation of Candidature Panel Recommendation Approval:

Panel Chair Name: Denny Meyer Signature: D. H. Meyer

FACULTY APPROVAL

This report is not final until signed by the Associate Dean, Research and Development (or designate)

The outcome of the Confirmation of Candidature is supported by the Faculty. ☐ Yes ☐ No

If no, what is the Faculty's recommendation?

Name: Signature:

CONFIRMATION OF CANDIDATURE REVIEW FORM
for Doctoral and Masters Candidates

SECTION F: ACTION PLAN
To be completed if Confirmation of Candidature Review is unsatisfactory

Where the Panel recommends that Confirmation of Candidature is unsatisfactory and not passed, the Candidate, in consultation with the Principal Supervisor, is required to develop an Action Plan within 10 business days of the first panel meeting. The Action Plan developed requires endorsement from the Confirmation of Candidature Panel Chair and must be reviewed at the follow-up meeting.

Please state clear, measurable goals to be completed by the time of the follow-up review that will ensure the student's progress is on track, both in terms of timeline to progress and in terms of the quality of the student's work. State what type of written submission is required and the submission(s) due date(s). Clearly state the scope, content, length and any other details required in the written document for the follow-up meeting. (Attach additional pages if necessary)

Action Plan approved by:

Candidate:

Principal Supervisor:

Review Panel Chair:

CONFIRMATION OF CANDIDATURE REVIEW FORM
for Doctoral and Masters Candidates

SECTION G: CONFIRMATION OF CANDIDATURE FOLLOW-UP REVIEW OUTCOME

Please indicate the Panel's recommendation:

☐ **Pass**
The Confirmation Panel recommends candidature be confirmed.

☐ **Candidature is not confirmed**

For doctoral candidates, the panel recommends an offer be made to the candidate to convert to Masters by Research. ☐ Yes

Please complete Section H of this form

Does the candidate have a stipend scholarship? ☐ Yes ☐ No

If yes, type of scholarship: --

Does the Panel recommend that the scholarship be continued? ☐ Yes ☐ No

☐ **Candidature is not confirmed.**

The panel recommends that candidature should be terminated.

Confirmation of Candidature Panel Recommendation Approval:

Panel Chair Name: Signature:

FACULTY APPROVAL
This report is not final until signed by the Associate Dean, Research and Development (or designate)

☐ Yes ☐ No The outcome of the Confirmation of Candidature is supported by the Faculty

If no, what is the Faculty's recommendation?

Name: Signature:

SECTION H: APPLICATION FOR CONVERSION

This section is to be used when recommending conversion between a PhD, Professional Doctorate or Masters by Research.

Current program: --

Conversion to: --

Graduate Studies

CONFIRMATION OF CANDIDATURE REVIEW FORM for Doctoral and Masters Candidates

SECTION H (i): Panel Recommendation

To be completed by the Chair of the Applicant's Confirmation of Candidature Panel.

Please provide a brief explanation of why you approve of this conversion.

SECTION H (ii): Supervisory Team Support

To be completed by the Principal Supervisor

Please provide a brief explanation of why you approve of this conversion.

Supervisory Team Composition:

Will there be any changes to the supervisory team? ☐ Yes ☐ No

If yes, the candidate must complete a Change of Supervisor form.

SECTION I: FACULTY APPROVAL OF CONVERSION

To be completed by the Associate Dean, Research and Development (or designate)

If the applicant is a stipend scholarship holder and converting from a Doctoral to a Masters program do you recommend that the stipend scholarship be continued? ☐ Yes ☐ No

Are you requesting an extension to the candidate's RTP Fees Offset funding or Tuition Fee Scholarship? ☐ Yes ☐ No

The Faculty recommends the application for conversion of candidature and certifies that the resources and supervisory support needed for the student's research program are available.

Program: --

Course Code: Unit Code:

Name: Signature:

CONFIRMATION OF CANDIDATURE REVIEW FORM for Doctoral and Masters Candidates

SECTION J: PVC (GRADUATE RESEARCH AND RESEARCH TRAINING) ENDORSEMENT

I endorse the requested course conversion. ☐ Yes ☐ No

Pro Vice-Chancellor (Graduate Research & Research Training):

Signature:
